# Distortion, confusion, and impasses: could a public dialogue within Knowledge Landscapes contribute to better communication and understanding of innovative knowledge?

**DOI:** 10.3325/cmj.2014.55.54

**Published:** 2014-02

**Authors:** Anna Lydia Svalastog, Joachim Allgaier, Lucia Martinelli, Srecko Gajovic

**Affiliations:** 1 Centre for Research Ethics and Bioethics, Uppsala University, Uppsala, Sweden; 2 Independent Scholar, Aachen, Germany; 3 MUSE – Science museum, Trento, Italy; 4 University of Zagreb School of Medicine, Croatian Institute for Brain Research, Zagreb, Croatia

Sustainable solutions and political regulation require public discussions, engagement, and dialogue at the interfaces between science and society. The communication and medialization of innovative knowledge is a key aspect for societal engagement, but also a major source of distortion. Knowledge about bio-objects, generated from biomedical and biotechnical research, causes uncertainty and societal controversies about ethics and risk, and new communicative strategies are required to face complex and potentially controversial topics generated by biology innovation, such as in vitro fertilization (IVF), stem cell research, gene therapy, (xeno-) transplantation, biobank research, recombinant pharmaceutics, etc. The amount of innovative knowledge is constantly increasing, and because of the internet, and Open Access initiatives, it is mostly freely available. However, the abundance of information available on the web, together with the fact that almost everybody can distribute information of varying quality on social online media, often leads to an overflow of communication and the situation of communication impasses. The delegation of the public communication of innovative scientific knowledge to public relations (PR) experts adds a further source of distortion in the communication process. Although communication aims at dialogue, this is frequently not achieved due to the variety of communicators involved and their different goals.

In contrast to former discussions focusing on dialogue between science, policy makers, and the public, the aim of this paper was to focus on the broader communicative situation related to the innovative knowledge in general and bio-objects in particular and to introduce the concept of Knowledge Landscapes to conceptually capture the present communicative situation. Knowledge Landscapes contain all the different communicators in these fields, the content communicated, and the pathways used for communication. We argue that today the Knowledge Landscapes appear as a confusing sites for gaining information and knowledge, reflecting current changes in the communication infrastructure, research, and political system and industry, and that we need physical and virtual communicative spaces where dialogue seeks to understand and critically discuss the content distributed in the Knowledge Landscapes. We also need to acknowledge that knowledge communication is vital for not only democratic advances and decision making processes, but it is also infused in the everyday life, delivering the democratic principles to all spheres of society, in particular to the field of medicine and health care, not the least due to the new paradigm referred to as personalized medicine. The presence and complexity of the scientific issues in the Knowledge Landscapes are in need of clarification and processing in an open-ended dialogue to gain a qualified perception of the Knowledge Landscape itself, and we need permanent sites for public reflection. An informed perception of Knowledge Landscapes and dialogue does not eradicate disagreement and controversy, but it is a prerequisite for a dynamic and democratic process as well as for dialogue itself. Science centers and museums serving as a local agora are proposed as one such potential arena, representing a safe space for difficult conversations where science and society can meet and engage in dialogue. The dialogue-based science communication should result in the benefits of citizens; make sure that decision making is legitimate; inform decision-makers about public opinion (support as well as resistance); and guarantee that dynamic and broad dialogues (processes) are established so that new and inventive strategies can be identified and developed.

## The need to communicate innovative knowledge

The amount of newly generated scientific information increases constantly. In parallel there is a general abundance of all kinds of information offered to citizens, which is enabled through advances in information technologies and the wide availability of data stored on the internet. Together with the increase in available information, there is an advance of knowledge within all disciplines. This newly generated knowledge is critical for the further development of our society. We refer to this subset of new knowledge as innovative knowledge, emphasizing its importance to maintain the vitality of society and contribute to knowledge driven economies. “Bio-objects” derive from innovative knowledge and are generated through the “bio-objectification” process continuously negotiated in the intersection of science, politics, and society (1,2). As such, their representation through communication is crucial (3). Bio-objects are defined as biological entities which escape classifications, and subsequently stir public responses to resolve this classification conflict. A suitable example is the classification of human embryos, which are results of in vitro fertilization processes, but left as a surplus of the procedure. Being stored in freezers across the world, they escape classification which would indicate how to handle them. It remains unclear whether they should be considered as human beings, or human samples, or biomaterial with economic value, or just biological waste. They could be used to generate new human beings, or human embryonic stem cells, they could be kept in the freezer forever, or be discarded. It is unclear whether they belong to the parents, to the hospital, or to the society. This example shows the contextually bound meaning of bio-objects, and a fracture between the application of new knowledge and society. As the governance of bio-objects depends on democratic mechanisms, this identification conflict cannot be solved among professionals and selected institutions alone (4). The stakeholders should already at early stages be involved in a public dialogue that facilitates responsible research and innovation (RRI), responding to societal needs, requirements, and desires (5).

## New knowledge-communication strategies

Knowledge generated from biomedical and biotechnical research is complex, and to gain legitimacy in decision making and governance questions, public acceptance is a key challenge in democratic societies. Different strategies are applied to involve the public, meet democratic challenges, and gain legitimate decisions and frameworks regarding development, distribution, and commercialization of innovative science and bio-objects (eg, the Eurobarometer surveys on science and technology) (6). In addition, sustainable solutions and political regulation are in need of thorough discussions to both collect and develop perspectives and strategies. As such, communication with the public is not a one way street but ideally a dynamic dialogue that produces new insights into the issues on the agenda. To establish dialogue at the interfaces between science and society, new arenas have been developed. For example, the Sciencewise Expert Resource Centre – the UK’s national center for public dialogue in policy making involving science and technology issues **(***http://www.sciencewise-erc.org.uk/*) was established in 2007. It exemplifies the political need and societal urge for a better and more dynamic and productive interaction between policymakers and the public to gain informed policy decisions involving science and technology issues. In this context, Mohr et al (7) emphasize how the public should be approached in plural, as publics, through dynamic dialogue where both interest groups and not yet involved individuals are invited on board, not only for the sake of democracy, but for the sake of collecting and developing a broad and relevant understanding of what is at stake and to identify possible solutions and strategies.

In this paper we launch the term Knowledge Landscapes and define it as the present communicative situation for innovative knowledge in general and bio-objects in particular, containing all the different communicators in these fields, the content communicated, and the pathways used for communication. We argue that today, Knowledge Landscapes appear as confusing sites for gaining information and knowledge, and we argue that there is a present need for arenas where dialogue on knowledge and innovative science and bio-objects can be established.

## Knowledge Landscape challenges

The abundance of information available on the web, together with the fact that almost everybody can distribute information of varying quality via online media (eg, social networks), often leads to an overflow of data and the situation of communication impasses. The need for dialogue and communication regarding innovative science, new technology, and bio-objects goes hand in hand with an urgent need to establish an open ended dialogue to gain a qualified perception of the knowledge. To achieve this we need to identify arenas where these issues can be discussed, not once or as a unique happening but as permanent sites for public reflection. The debate is needed not for policy reasons, but because of the presence and complexity of the scientific issues in a need of clarification and processing. We therefore argue for the creation of communicative spaces where the consequences of innovative knowledge could be discussed without the distortions mentioned before. In such a Knowledge Landscape actors and stakeholders from various arenas (science, the public, industry, politics etc) should be able to meet and discuss freely and equally. In this context, societal dialogue will imply epistemological clarifications, ie, clarification of how scientific knowledge (not only scientific results) is constructed. This will produce a transparent communication that will avoid misunderstanding and confusion, and make a dialogue a joint interpretative project. Hermeneutical ideals from both Habermas and Gadamer underline the fundamental importance of communication and the necessity of reciprocal understanding for conversation, dialogue, and interpretation to be real/true (in contrast to untrue, ie, represent misunderstandings and confusion) (8,9). Science centers and museums serving as a local agora are here proposed as one such potential space where science and society could meet and engage in true dialogue. However, current changes in the communication infrastructure, industry, research, and political system require the creation of more such physical and virtual spaces where the results and consequences of innovative knowledge can be discussed between scientists and other members of society.

## Knowledge Landscapes and the “medialization” of science

A central topic in the scholarly investigation of science-society relationship is the “medialization” of science (10). Medialization means that science increasingly comes under pressure to publicly legitimize itself, which results in an increasing orientation on the public communication of science. Due to more public scrutiny, science is held responsible and accountable, eg, for public research funds it receives. This process comes into play when researchers compete for resources, visibility, and public attention with one another, but also with other subject areas and actors. In this situation most universities and research organizations employ public relations (PR) experts to professionalize and manage their interactions with various audiences, but also especially with the mass media. As the degree of the medialization of science and research increases, the more important becomes the role of public relations management for the scientific institutions (11). This also has an influence on *how* and *on what terms* innovative scientific knowledge is publicized. The most central aim of science PR is the public legitimization of the research organization to secure the resources that the organization needs. To do so, they have to demonstrate usefulness, excellence, and public support to their funders and society as a whole. In this sense, public relations management is seen as an instrument to secure the autonomy of scientific organizations.

However, this development has some unintended side-effects. In the context of PR work, the meaning and value of scientific knowledge can change. For instance, a basic researcher in the neurosciences stressed that the chances for getting public attention increase if a potential medical applicability of their research is mentioned in press releases: “…it depends on the audience, but in press releases it [our research] is always, always disease-relevant” (12).

If the innovative knowledge about bio-objects is communicated with the help of PR experts, the meaning of the knowledge may be transformed and often value is added. This is most often the case when potential medical or other applications are stressed in press releases. The “marketing” of knowledge in that way can lead to false hopes or unfounded fears, but also to ill-informed decisions on matters of health (13).

These applications stressed in press releases were not necessarily of interest for the scientists who conducted the research in the first place or even the cause of basic research. Societal and also economic values are ascribed to the innovative knowledge that leads to applications or patents, also to “sell” the knowledge to other actors than scientists. It is likely that different actors and stakeholders ascribe different meanings, values, and intentions to the innovative knowledge coming from biotechnological research. This process of adding potential value to the innovative knowledge in many cases creates distortions to the original knowledge, as the potential value starts to be more important than the actual content. In addition, skillful PR can add desired values to any content, entering in the marketing battle, which adds to the uncertainty which piece of knowledge is really relevant to the user, citizen, or patient.

## Scenarios of innovative knowledge communication

Conferences and workshops, journals, books, seminars, and lectures are common ways of communicating new knowledge in the scientific community. The public is usually excluded here. New options for communications, which have become more and more important, are Open Access journals and public events at universities and research institutions, suited for various publics. Further mediated distribution of scientific knowledge is done through popularized books and lectures, TV-programs, print media, and radio, which enables non specialists to consume and learn about innovative knowledge in their spare time and in their private life. Further complexity is added by the nature of social online media, such as social online networks (for example Facebook), and blogs and micro-blogs (eg, Twitter). The social online-video network YouTube is a particularly prominent example. It is a useful tool for scientific knowledge dissemination (eg, lectures, interviews etc.), but at the same time it is also used by amateurs or interest groups (including patient groups, religious groups, and political sub-groups etc) to promote their ideas and views. On YouTube all contributors are presented equally, little or no censorship is taking place, and there is no inwards hierarchy regarding scientific accuracy and validity of the content. For example, concerning stem cells, one will find videos of knowledgeable scientists, side by side to videos promoting unapproved experimental stem cell-treatments that can be risky and harmful for patients.

The combination of research, marketing, conspiracy, speculation, and wrongful assumptions, which can be found on the web, should be perceived as such: a mixture of incompatible and contradictory messages distributed through Open Knowledge Distribution. Receiving information, though one can sometimes communicate through feedback or chat options, is different from educational settings where conversation and dialogue facilitate tools that help the individual elaborate, understand, and integrate new knowledge, as well as recognize which possibilities and limitation this knowledge entails.

At first sight, Open Knowledge Distribution is a positive development providing information to everyone. It is an important pathway for disseminating knowledge, but also, as described above, a problem. Regarding bio-objects, Open Knowledge Distribution contributes to bio-objectification processes. The results though, can represent both confusion and misunderstandings, leading to controversies about bio-objects that represent new challenges and require a new quality of communication, where the establishment of communicating arenas is a given priority.

The new communication quality implies insights into the actors, content, agendas, and communication pathways in the Knowledge Landscapes. The Knowledge Landscapes in the future should contain a variety of overlapping arenas (eg, academic, cultural educational and governing institutions, industry, media, NGOs, interest groups, homepages of individual agents etc.), where a variety of communicative pathways are established and new ones continuously develop. The different arenas in the Knowledge Landscapes are not strictly separated, and the overlaps make it necessary to approach communication in this landscape as a whole. Described in terms of a landscape, the communication paths for bio-objects constitute a complex and varied scenario where dissemination of knowledge is sent and received, discussed, used, and consumed by individuals both inside and outside of academic institutions. What we suggest is to push forward the need for a dialogue that includes the public, and aims at establishing a continuous and dynamic dialogue that seeks to understand and critically discusses the content distributed in the Knowledge Landscapes. If such a dialogue succeeds, the Knowledge Landscapes will no longer appear as a bare chaos or anarchy (arrows being shot out more or less blindly) but as representing interrelated dynamic processes of communication.

An informed perception of Knowledge Landscapes and dialogue does not eradicate disagreement and controversy, but it is a prerequisite for a dynamic and democratic process as well as for dialogue itself. Ideally, a Knowledge Landscape represents a dynamic area where experts and various publics can express their heterogeneous natures and interact, evolve, exchange, and change perspectives in inclusive dialogues (7,14).

Because the communicative pathways of bio-objects are many, overlapping and interacting, the Knowledge Landscape of communicative pathways can be difficult to grasp. If the publics are to get hold on the communicative pathways a prerequisite is transparency. Due to strategic and political implications, this might not always be possible as sometimes some actors will not be willing to participate. Because of the political and strategic implications, and due to the complexity of the dynamics involved in the communication of bio-objects, there is a need for qualified research, innovative applications, and individuals who have the time and competence to organize and analyze the complexity of the communicative landscape of innovative knowledge.

## Bio-objects and communication issues

Currently there are many ways to publish and share the information concerning the innovative knowledge, resulting in an information overflow. Although information is widely available for free and is abundant, selecting and applying the relevant knowledge may become quite difficult and includes public controversies regarding priorities, ethics, and risk handling, in addition to the fact that scientists themselves represent different agendas, viewpoints, and policies. We suggest that the complex interrelations and dynamics of the Knowledge Landscapes, the complexity of the issues presented and discussed above, together with the discrepancy in the generation of innovative knowledge disrupt dialogue and result in novel societal phenomena. The process of bio-objectification is an illuminating example of the communication impasse generating new issues, including the need for arenas for communication and debates. Bio-objects are distributed but also identified, reshaped, and invented in the Knowledge Landscapes, taking the form of information, policy, politics, critique, rumors as well as “urban myths.” Moreover, we suggest that the inappropriate communication of knowledge is implicated in the generation of the controversies concerning bio-objects and therefore has a relevant role in the bio-objectification process itself.

Although the innovative knowledge is needed and indeed generated, the wide availability of data alone does not correspond to the wide availability of knowledge. The new technologies and new approaches require a specialized skillset to understand and apply the generated innovation. Therefore, we face a paradox of widely available information including also the innovative knowledge, but still those having the skillset to identify and use the knowledge are rare. As a consequence there is a combination of relevant and misguided doubts and fears about bio-technological and biomedical innovation because of an information overload in the Knowledge Landscapes. The complexity embedded in new knowledge includes questions regarding priorities, ethics, and risk handling, implementation of the new knowledge in clinical settings, industry, agriculture, policies, and politics etc. The application of innovative knowledge turns out to be challenging and controversial, represents conflicts of interests and is in need of dialogue to clarify misunderstandings and unnecessary delays in transferring the new knowledge into technologies to the benefits of citizens; make sure that decision making is legitimate; inform decision-makers about public opinion (support as well as resistance); guarantee that dynamic and broad dialogues (processes) are established, so that new and inventive strategies can be identified and developed.

## Museums as an agora of communication

Among the various arenas for the communication of science innovation, science museums and science centers are important institutions where visitors can learn, play, talk, and think. Exhibits offer to citizens of every age opportunities for learning science with a social dimension as well as with the relevant content knowledge (15). Creating scientific citizenship, where every citizen has rights and responsibilities related to the knowledge distribution and usage, is a crucial component of museums’ new mission. Achieving biological- and biomedical-citizenships would enable citizens to acquire the necessary capability to face the complex issues generated by biology and biomedical innovations, and thus enable them to navigate in the Knowledge Landscapes (16).

Bio-objects, with their overload of social issues, are suitable topics to be proposed to the public in museums and science centers. The possible formats to use are permanent and temporary exhibitions as well as new tools for contemporary scientific museology (science-theater, open-door laboratories, nights at the museum, scientific cafes, and “Science in the Street” events), which are open to influences from literature, philosophy, social sciences and arts (17). Though reflecting societal events, trends, and needs, neither of these options of present museology is restricted to the task of clarifying issues of acute urgency to policymakers. Accordingly, science museums of new conception, in addition to their mission to promote the public understanding and engagement on major conceptual advances in the natural and life sciences, are becoming recognized “*agora*,” ie, central meeting points where all can engage in science innovations, learn, and share expertise and experience. Their event programs are increasingly focusing on debates, dialogues, and public interactions with science and society related aspects, rather than the more traditional public lecture format. In addition, on the basis of their networks, museums can reach large numbers of people on a physical territory but also in virtual spaces, and thus become a vital arena for engaging various publics and local communities in a dialogue about innovative knowledge. As already stated by the Toronto Declaration signed in 2008 by four-hundred science centers and museums across the world, museums present themselves to society as “safe places for difficult conversations,” ie, “places where controversial issues related to the ethical, social and economic impact of research and of new technologies can be discussed in an open and informed way” (18,19). Museums are arenas where knowledge transfer and dialogue can take place between those producing innovative knowledge (eg, scientists and researchers) and those using it (eg, enterprises, policy makers, citizens) (20). The direct contact among stakeholders could also avoid the distortions, which are often the result of mediated communications. In addition to this general goal, we want to encourage museums to explicitly address challenges related to the complexity of the Knowledge Landscapes.

## Knowledge Landscapes and personalized medicine

In today’s globalized and individualized society, the competence of critical thinking is a key issue for citizen, and for society it should be a key task to facilitate arenas where this competence can be acquired and continuously updated and developed. This should enable them to decipher the complex situation of the Knowledge Landscapes, where content and communication paths can be contextualized, premises and interrelations can be made visible, and content can be more properly understood. This task is important because knowledge communication is vital for, not only democratic advances, and decision making processes, but it is also infused in the everyday life, delivering the democratic principles to all spheres of society. This knowledge-based democratization applies in particular to the field of medicine and health care. A new paradigm referred to as personalized medicine appears to be a highlight of the new technologically based health system. If properly communicated, the medical knowledge stops to be a privilege of the professionals, turning into a tool for those needing help. The idea of this new system of personalized medicine is to include the patient in decision-making processes (patient-centered medicine), contributing to the diagnosis and the treatment of the patient as a whole (person-centered medicine). Personalized medicine does not downgrade the need for professional expertise, which would indeed still be the responsibility of the specialized experts, but empowers the patients to pave their ways to health and well-being and subsequently to make responsible choices in managing their lives. This dialogue-based landscape of personalized medicine is another example of the necessary improvement of our communication modalities, and it underlines the importance of a proper and critical understanding of innovative and biomedical knowledge (21).

## Conclusion

The importance of appropriate and shared communication within Knowledge Landscapes and the necessity of engaging citizens in all phases of their life in a dialogue are indispensable tools needed for the functioning and advancement of today’s society. To achieve this, particular care should be given by all stakeholders to be involved in the dialogue, and by society to facilitate arenas within Knowledge Landscapes. This should enable the flow and exchange of knowledge and its use for the benefit of economy, health system, democracy, and society in general.
